# Transcriptomic and metabolomic analysis of carotenoid metabolism and accumulation of carotenoid-derived products in tobacco leaves before and after curing

**DOI:** 10.3389/fpls.2025.1671379

**Published:** 2025-10-10

**Authors:** Tao Liu, Na Kong, Jianmin Cao, Gang Chen, Zhichao Deng, Lichao Wen, Zhenbiao Zhang, Cun Guo, Rongrong Wu, Yalun Yang, Wei Li, Yongfeng Guo

**Affiliations:** ^1^ Qingdao Municipal Key Laboratory of Plant Molecular Pharming, Tobacco Research Institute, Chinese Academy of Agricultural Sciences, Qingdao, China; ^2^ College of Biology and Food Engineering, Chongqing Three Gorges University, Chongqing, China; ^3^ College of Agriculture, Tarim University, Alar, China

**Keywords:** fresh tobacco leaves, flue-cured tobacco leaves, transcriptome, carotenoid degradation products, aroma substances

## Abstract

As an important cash crop, tobacco (*Nicotiana tabacum L*.) is grown to harvest leaves which are cured at elevated temperature before sale. The quality of cured leaves impacts the economic return of growers. High-quality tobacco has high levels of aroma substances in cured leaves, derived mainly from carotenoid degradation products (CDPs). Significant difference in CDPs levels occurs between varieties of distinct genetic background and between regions with different ecological conditions. In order to have a better understanding of what are the major factors that influence the accumulation of CDPs in tobacco leaves, four tobacco varieties, K326, NC82, G28, and Gexin3, with different aroma characteristics, were grown in two typical fields in Shandong and Yunnan, two major tobacco-producing areas in northern and southern China, respectively. Transcriptomic and metabolomic analysis was conducted to identify differentially expressed genes and metabolites. The results of transcriptome analysis revealed that the number of differentially expressed genes (DEGs) between the two locations was more than three times of that among varieties, with the DEGs between locations enriched in the carotenoid biosynthetic pathway. For fresh leaves, similar group of carotenoid-related DEGs were identified and more significant difference in carotenoid contents was observed between locations than among varieties, with higher carotenoid accumulation in tobacco grown in Shandong. Significant decrease in carotenoid contents in leaves was observed during the curing process after harvest. For the same variety, the reduction in carotenoid content following curing was greater in Shandong than in Yunnan. The accumulation of CDPs in tobacco leaves after curing, however, appeared to be complex, being influenced by both environmental and genetic factors. Among the detected CDPs, acetoin, geranyl acetone, and megastigmatrienone were primarily affected by environmental factors, whereas β-ionone-5,6-epoxyde and 3-oxo-α-ionol were predominantly influenced by genetic factors. This study systematically reveals the cascade regulatory relationship among “gene expression – carotenoid accumulation – aroma formation”.

## 1 Introduction

Tobacco (*Nicotiana tabacum* L.), as an important agricultural crop, is widely cultivated and consumed worldwide (Lewis et al., 2020). Mature tobacco leaves are harvested and are subsequently cured at elevated temperature before industrial use (Chen et al., 2019). Since cured tobacco leaves are often graded based on quality before sale, improvement of leaf quality is the major goal of tobacco breeding, especially in China, the largest tobacco consuming country. Leaf quality of tobacco is a complex trait consisting of physical appearance, chemical composition, and sensory evaluation results of cured leaves (Yao et al., 2024). It has been demonstrated that tobacco leaf quality can be affected by climatic factors, fertilization conditions and soil microorganisms at the field stage (Jiang et al., 2022; Li et al., 2021; Tang et al., 2020; Yan et al., 2020). At the harvest stage, tobacco quality is influenced by factors such as leaf age and harvesting methods (Chen et al., 2019; Meng et al., 2022). The quality of tobacco leaves is also influenced by curing conditions after harvest (Chen et al., 2021).

A plethora of evidence indicates that tobacco leaf quality, especially sensory quality properties such as aroma and taste, are dependent on the accumulation of a variety of secondary metabolites, including alkaloids, carotenoids, carotenoid degradation products (CDPs), amino acids, and polyphenols (Cai et al., 2016; Fritz et al., 2006; Popova et al., 2019; Tsaballa et al., 2020; Wen et al., 2023). Among these, carotenoids and CDPs are especially important due to their significant contribution to aroma and overall leaf quality (Popova et al., 2019).

Carotenoids, as C40 terpenoids consisting of an isoprenoid backbone, are the general term for a group of important fat-soluble pigments (Quian-Ulloa and Stange, 2021). A number of carotenoid components had been identified, such as β-carotene, lutein, violaxanthin, and neoxanthin, among others (Karppinen et al., 2016). For tobacco, carotenoids are not only an important class of secondary metabolites essential for its growth and development, but also a key factor determining the quality of flue-cured tobacco. As plastid pigments, they contribute to the golden-yellow color of tobacco leaves after curing (Guo et al., 2009). As significant aroma precursors in tobacco, their degradation produces various compounds with distinct and pleasant aromas, closely related to the aroma characteristics and intensity of tobacco leaves (Yan et al., 2014). Studies had shown a positive correlation between carotenoid content and tobacco leaf quality: tobacco leaves with higher levels of aromatic components tend to contain more carotenoids, which in turn corresponds to higher aroma quality in flue-cured tobacco (Xiang et al., 2024).

The metabolic pathway of carotenoids involves various genes, such as *geranyl diphosphate synthase* (*GGPPS*), *phytoene synthase* (*PSY*) and *phytoene desaturase* (*PDS*), and others (Nisar et al., 2015). In plants, the expression of these genes is regulated—directly or indirectly—by transcription factors. For instance, AtHY5 and NtDREB-1BL1 promote carotenoid biosynthesis by binding to the *PSY* promoter and enhancing its expression (Dong et al., 2022; Toledo-Ortiz et al., 2014), whereas SlPIF1 inhibits *PSY* expression (Llorente et al., 2016). Some of the transcription factors are capable of influencing the expression of multiple genes in the carotenoid metabolic pathway simultaneously, such as SlBZR1, SlPRE2 and SlAP2a (Chung et al., 2010; Karlova et al., 2011; Liu et al., 2014; Zhu et al., 2017).

The concentration of CDPs in cured tobacco has a direct impact on aroma, taste and overall quality of tobacco leaves. During growth, curing, and the aging process after curing, enzymatic and non-enzymatic reactions can result in the degradation of carotenoids in tobacco leaves, leading to the production of aroma substances. The two primary enzymatic pathways are mediated by carotenoid cleavage dioxygenases (CCDs) and lipoxygenases (LOXs). CCDs are non-heme iron proteins that catalyze the oxidative cleavage of carotenoids to yield apocarotenoids (Daruwalla and Kiser, 2020), while LOXs peroxidize carotenoids and polyunsaturated fatty acids to generate volatile aromas (Nisar et al., 2015). Enzymatic cleavage of carotenoids catalyzed by members of the CCDs family has been shown to produce a number of industrially important volatile flavor and aroma apocarotenoids including β-ionone, geranylacetone, pseudoionone, α-ionone and 3-hydroxy-β-ionone in a range of plant species (Lashbrooke et al., 2013). LOXs mediate a co-oxidation reaction that results in the nonspecific cleavage of carotenoids and produces apocarotenoid volatiles, such as β-ionone, β-cyclocitral, β-ionone-5,6-epoxide, and dihydroactinidiolide (Liang et al., 2021). Non-enzymatic degradation occurs primarily during the late stages of curing, with the increase in temperature leading to carbon chain breaks and the generation of aroma substances. Over a hundred CDPs have been identified in tobacco (Wahlberg, 2001), the majority of which possess appealing aromas. These include β-ionone, which exudes a violet scent (Aloum et al., 2020), geranyl acetone, which evokes a magnolia aroma (Bonikowski et al., 2015) and megastigmatrienone, which emits tobacco-like aromas (Slaghenaufi et al., 2016). In addition to exhibiting low aroma thresholds and excellent aroma, CDPs have been demonstrated to mellow the smoke and reduce irritation (Weng et al., 2024). These aroma substances can also be directly added into tobacco as additives to improve tobacco quality. For instance, the addition of megastigmatrienone or β-ionone to tobacco significantly enhances the aroma quality and intensity of cured tobacco leaves, while reducing harshness and irritancy (Ji et al., 2011; Liu et al., 2022).

Previous studies have covered the mechanisms of tobacco aroma substance formation and metabolite regulatory networks through transcriptomic, proteomic, and metabolomic analyses (Liu et al., 2022a, 2022b; Liu et al., 2020). However, most of these studies focused on fresh tobacco leaves and failed to establish a link with metabolic changes in tobacco leaves after curing, which is the ultimate expression of tobacco leaf quality. In this study, we attempted to reveal mechanisms of the formation of CDPs in cured tobacco leaves from both environmental and genetic perspectives, with the aim of providing novel insights for enhancing the aroma quality of tobacco leaves.

## 2 Materials and methods

### 2.1 Materials and sample preparation

In 2021, tobacco (*Nicotiana tabacum* L.) varieties K326, NC82, G28, and Gexin3 were planted in Taoxu Town (35°37′26″N, 118°01′22″E), Linyi City, Shandong Province, and Xinjie Town (25°22′22″N, 100°27′31″E), Dali City, Yunnan Province, with the objective of collecting fresh and cured tobacco leaves (**Supplementary Figure S1**). A field with uniform soil fertility and flat terrain was selected. A randomized block design was adopted with three replicates. Each plot consisted of three rows with a total of 120 plants, spaced at 1 m between rows and 0.5 m between plants. Guard rows were established. Field management strictly followed local production practices, and all agricultural operations were consistent across plots.

For fresh leaf samples, 70 days after transplanting, leaves from the middle portions (the sixth to eighth leaves from the bottom) were harvested. Three biological replicates were collected with each replicate containing four half leaves. Each harvested leaf was bisected immediately along the main vein into two equal portions. One half was utilized for the determination of carotenoids and aroma substances, while the remaining half was used for transcriptomic analysis. Harvested leaves were frozen with liquid nitrogen and stored at -80°C.

For cured leaf samples, mature leaves from the middle positions were subjected to a three-stage curing process (**Supplementary Figure S2**). Subsequently, the cured tobacco leaf samples were used for analysis of carotenoids and aroma substances. Three biological replicates were conducted for each treatment.

### 2.2 RNA extraction and transcriptome sequencing

Total RNA was extracted following the previously described method (He and Gan, 2002). The RNA sample integrity and concentration were assessed using an Agilent 2100 bioanalyzer. The experimental procedures for mRNA purification, library construction, and transcriptome sequencing were conducted by Novogene Bioinformatics Technology Co., Ltd. (Tianjin, China). For individual samples, mRNA was captured using Oligo (dT)-conjugated magnetic beads and sequentially purified with binding and washing buffers. The isolated mRNA underwent fragmentation into 100~200 nucleotide fragments using a fragmentation buffer, followed by reverse transcription to generate cDNA. The synthesized cDNA was recovered via DNA Clean Beads and subsequently ligated to UMI-containing adaptors in a single-step PCR reaction utilizing ligase and buffer. Post-ligation products were further purified with DNA Clean Beads and amplified by PCR. Amplified DNA fragments were purified using the same beads and eluted in nuclease-free water. After library preparation and pooling, sequencing was performed on the Illumina platform.

### 2.3 RNA-seq data analysis

Raw sequencing data in FASTQ format were first analyzed through in-house Perl scripts to generate high-quality reads. A reference genome index for *N. tabacum* variety K326 (Nitab-v4.5_Edwards2017) was built with HISAT2 (version 2.0.4), followed by alignment of paired-end clean reads to the reference genome using the same software. To identify differentially expressed genes between experimental groups, the DESeq R package (version 1.10.1) was utilized, which applies a negative binomial distribution model to assess statistical significance. The Benjamini and Hochberg procedure were employed to adjust raw P-values, thereby controlling the false discovery rate. Genes exhibiting adjusted P-values below 0.05 were classified as differentially expressed.

### 2.4 Real-time PCR validation

For RT-qPCR analysis, total RNA (1 μg) was reverse-transcribed into complementary DNA using the HiScript III All-in-one RT SuperMix Perfect for qPCR kit (Vazyme, China). Gene expression profiling was subsequently performed using the ChamQ Universal SYBR qPCR Master Mix (Vazyme, China) on a 7500 real-time PCR system(Applied Biosystems). The thermal cycling parameters consisted of an initial denaturation at 95°C for 30 sec, followed by 40 cycles of denaturation at 95°C for 10 sec and combined annealing at 60°C for 30 sec. The tobacco NtActin7 gene was used as the internal reference. Primer sequences employed in this study are comprehensively detailed in **Supplementary Table S8**.

### 2.5 Carotenoid extraction and LC-MS/MS analysis

The samples were lyophilized for 10 hours in the freeze-dryer. Lyophilized samples were pulverized into fine powder and stored at -80°C prior to analysis. For carotenoid extraction, 50 mg of the powdered material was extracted with 0.5 mL of mixture containing n-hexane, acetone, and ethanol (1:1:1, v/v/v). Filtered through 0.22 μm membranes, the extracts were analyzed via LC-MS/MS using an UPLC-APCI-MS/MS system (UPLC, ExionLC™ AD; MS, Applied Biosystems 6500 Triple Quadrupole) (Bartley and Scolnik, 1995; Inbaraj et al., 2008). The liquid chromatography (LC) used an YMC C30 column (100 × 2.0 mm, 3 μm) with mobile phase A (methanol/acetonitrile, 1:3 v/v, 0.01% BHT, 0.1% formic acid) and mobile phase B (methyl tert-butyl ether, 0.01% BHT). The gradient protocol proceeded as follows: 0% B (0–3 min), 70% B (3–5 min), 95% B (5–9 min), and 0% B (10–11 min). Operating parameters included a flow rate of 0.8 mL/min, column temperature of 28°C, and injection volume of 2 μL. Carotenoid quantification was conducted via scheduled multiple reaction monitoring (MRM) with the Analyst 1.6.3 software for data acquisition. The Multiquant 3.0.3 software (Sciex) was used to quantify all metabolites (Geyer et al., 2004).

### 2.6 Analysis of aroma substances and GC-MS conditions

Samples for GC/MS analysis were prepared using headspace solid-phase microextraction (HS-SPME). Tobacco leaf samples were cryogenically ground with liquid nitrogen, and 0.5 g sample was transferred into 20 mL headspace vials fitted with polytetrafluoroethylene septa. Phenethyl ethyl acetate (102 μg/mL), serving as the internal standard, was introduced as a 1 μL aliquot into each vial via microsyringe injection. The vials were equilibrated on a temperature-controlled SPME platform. The extraction head, after being inserted into the headspace bottle for a period of time, was removed and immediately placed into the gas chromatograph inlet. Desorption was then performed at 250°C for 10 min, followed by GC/MS detection.

GC-MS analysis was conducted using a model 7890A gas chromatograph equipped with an HP-FFAP capillary column (50 m × 0.32 mm × 0.50 μm) and coupled to an Agilent 5975C mass-selective detector. The detector was operated in the splitless mode with electron impact ionization at an ionizing voltage of 70 eV. The carrier gas (99.9996% helium) flow rate was maintained at 1.5 mL min^-1^. The initial temperature was set to 50°C and held for 1 min, followed by a ramp at 10°C min^-1^ to 130°C. Subsequently, the temperature was increased at a rate of 3°C min^-1^ to 200°C, and then at 5°C min^-1^ to 230°C, with holding this temperature for 20 min. The ion source was maintained at 230°C. The transfer line was maintained at 250°C. The scan mode was employed for the MS detector, with a mass range of 33–600 amu.

### 2.7 Statistical analysis

Plotting bar graphs was performed using Prism 9 version 9.5.1. ANOVA was performed using Prism version 9, and differences among treatments were defined at *P* < 0.05. A heat map analysis was conducted with TBtools version 2.001 to visualize the relative levels of genes. All the images were processed using Adobe illustrator CC 2019.

## 3 Results and discussion

### 3.1 Transcriptome analysis of fresh tobacco leaves

**Figure 1:**
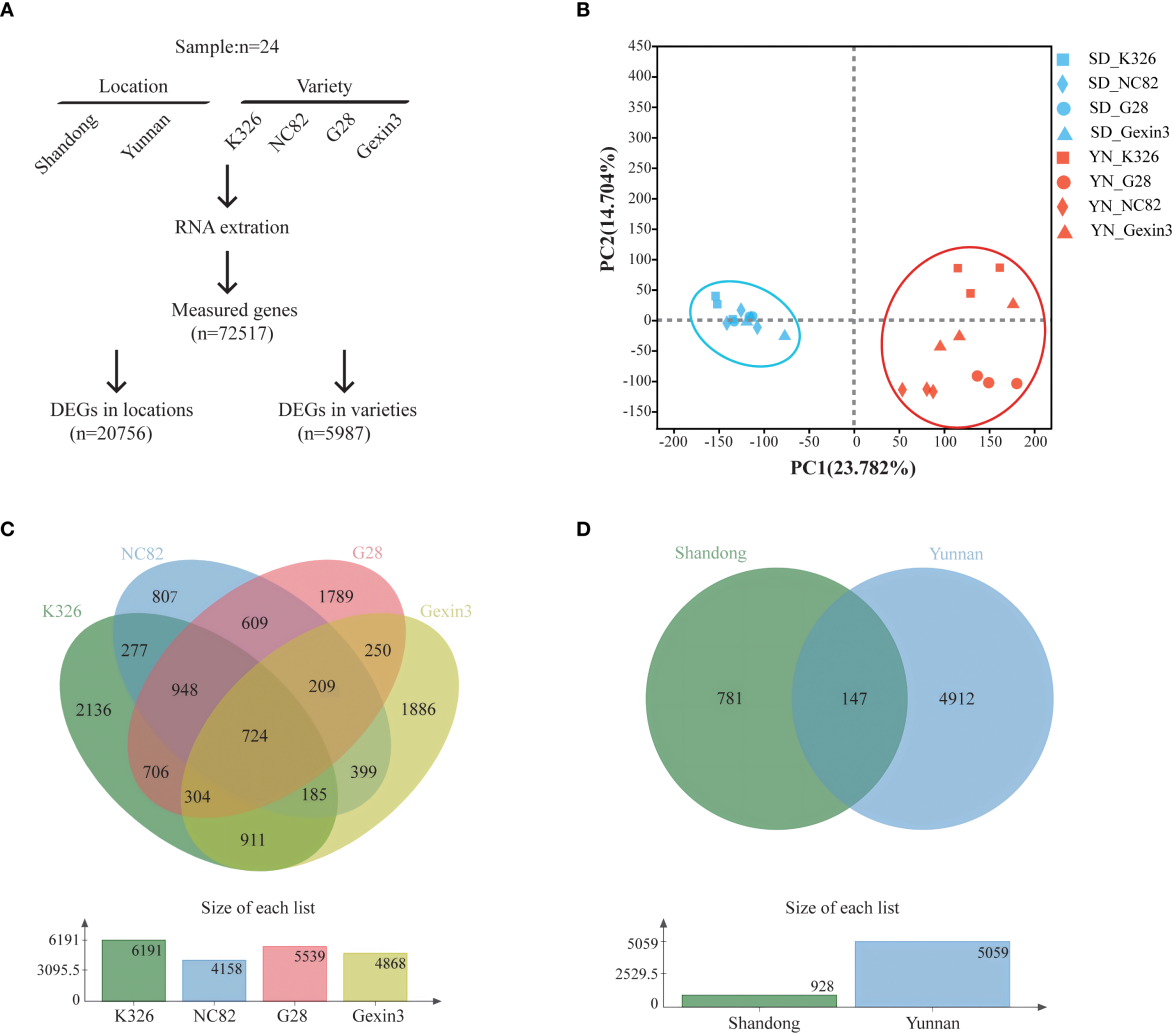
Transcriptome analysis. **(A)** Diagram showing the identification of differentially expressed genes; **(B)** Principal component analysis (PCA) of the transcriptomic data; **(C)** Venn diagram of DEGs in locations; **(D)** Venn diagram of DEGs in varieties.

To understand the influence of environmental and genetic factors on tobacco carotenoid metabolism, we planted tobacco varieties K326, NC82, G28, and Gexin3 in Shandong and Yunnan, which are representative tobacco producing regions in China. The tobacco varieties have distinct aroma styles. A total of 24 samples of fresh tobacco leaves were collected for RNA-seq analysis. Following quality control, approximately 30 million clean reads were obtained for each sample. Gene expression levels were quantified by comparison with the tobacco reference genome, resulting in the detection of 72,517 expressed genes (**Figure 1A**). The results of principal component analysis (PCA) demonstrated a discernible clustering of the two locations (**Figure 1B**) and a clear separation of the four varieties (**Supplementary Figure S3**), indicating the high quality of the RNA-seq data. By employing ANOVA and multiple test correction, we identified 5,987 differentially expressed genes (DEGs) among the four varieties (**Figure 1C**) and 20,756 DEGs between the two locations (**Figure 1D**) (|log2fold change|>2, *P* < 0.05). The number of DEGs between locations is more than three times of the DEGs among varieties, indicating that environmental factors exert a stronger influence on tobacco gene expression than genetic factors. This finding is consistent with a previous study, which demonstrated that more DEGs were shared between growing locations and developmental stages than among varieties in tobacco (Liu et al., 2020). Among the four varieties, gene expression of K326 exhibited the greatest changes caused by environmental factors, with 6,191 DEGs, while the least environmental impact was observed on gene expression of NC82, with 4,158 DEGs. The majority of the DEGs among varieties were from the Yunnan samples (5,059/5,987; 84.50%). These findings suggest that the growth environment in Yunnan is more conducive for the four varieties to display their distinctive characteristics.

### 3.2 GO and KEGG analysis of DEGs in fresh leaves

**Figure 2:**
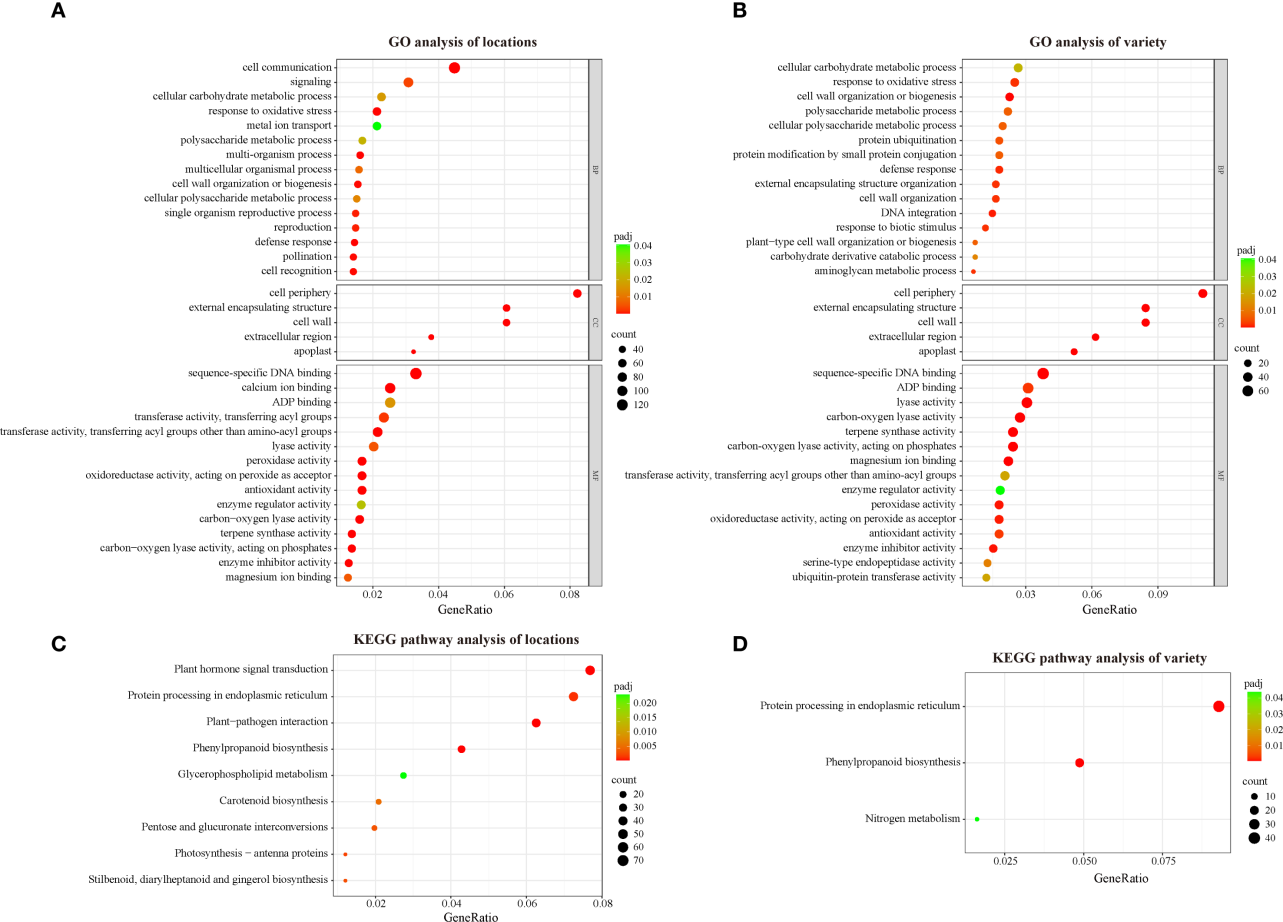
GO and KEGG analysis of DEGs between locations and among varieties. **(A)** GO analysis of location DEGs; **(B)** GO analysis of variety DEGs; **(C)** KEGG analysis of location DEGs; **(D)** KEGG analysis of variety DEG.

GO (Gene Ontology) enrichment analysis is a commonly used method for gene function annotation that can help us to understand the characteristics and functions of differentially expressed genes in terms of biological processes, cellular components and molecular functions (Gene Ontology Consortium, 2015). To understand the biological functions of the DEGs identified in this study, we performed GO enrichment analysis. The DEGs between locations (location DEGs) were annotated in 39 biological processes, 5 cellular components and 42 molecular functions (**Supplementary Table S1**). The major enriched biological processes in location DEGs include cell communication, signaling, and cellular carbohydrate metabolic process. The major enriched cellular components of location DEGs are cell periphery, cell wall, and external encapsulating structure. The major molecular function aspects enriched in location DEGs are sequence-specific DNA binding, calcium ion binding, and ADP binding (**Figure 2A**). The DEGs among varieties (variety DEGs) were enriched in 31 biological processes, including cellular carbohydrate metabolic process, response to oxidative stress, and cell wall organization or biogenesis. Five cellular components including cell periphery, cell wall, external encapsulating structure, and 39 molecular functions including sequence-specific DNA binding, ADP binding, lyase activity are enriched in the variety DEGs (**Supplementary Table S1**) (**Figure 2B**). Interestingly, identical GO terms were enriched at the cellular component level between the two sets of DEGs. Furthermore, a high degree of similarity was observed in terms of molecular function, with 13 of the 15 main GO terms being identical between the location DEGs and the variety DEGs. For the location DEGs, 131 and 90 DEGs are enriched in cell communication (GO:0007154) and signaling (GO:0023052), respectively. In contrast, no enrichment of these two terms was observed in the variety DEGs, which suggests that changes in the external environment could have a significant impact on signaling in tobacco.

Additionally, KEGG (Kyoto Encyclopedia of Genes and Genomes) enrichment analysis was employed to classify the DEGs and reveal their potential functions. KEGG is an effort to link genomic information with higher order functional information by computerizing current knowledge on cellular processes and by standardizing gene annotations (Kanehisa and Goto, 2000). Plant hormone signal transduction, protein processing in endoplasmic reticulum, plant−pathogen interaction, phenylpropanoid biosynthesis, glycerophospholipid metabolism, carotenoid biosynthesis, pentose and glucuronate interconversions, photosynthesis-antenna proteins and stilbenoid (diarylheptanoid and gingerol biosynthesis) are significantly enriched in location DEGs (**Figure 2C**, **Supplementary Table S2**), while only protein processing in endoplasmic reticulum, phenylpropanoid biosynthesis and nitrogen metabolism are significantly enriched in variety DEGs (**Figure 2D**, **Supplementary Table S2**). Notably, carotenoid biosynthesis is significantly enriched in location DEGs, suggesting that changes in environmental conditions could affect genes related to carotenoid biosynthesis in tobacco. As important aroma precursors in tobacco, carotenoids are degraded to a variety of small-molecule aroma substances during the curing process (Popova et al., 2019). Therefore, variations in the content in fresh tobacco leaves can affect the changes in a variety of aroma substances in cured tobacco leaves, which in turn affects the sensory quality. To further understand the changes of genes and related transcription factors related to the carotenoid biosynthesis pathway, we next verified the expression of these genes via qRT-PCR.

### 3.3 Expression changes of genes involved in the carotenoid biosynthesis pathway

**Figure 3:**
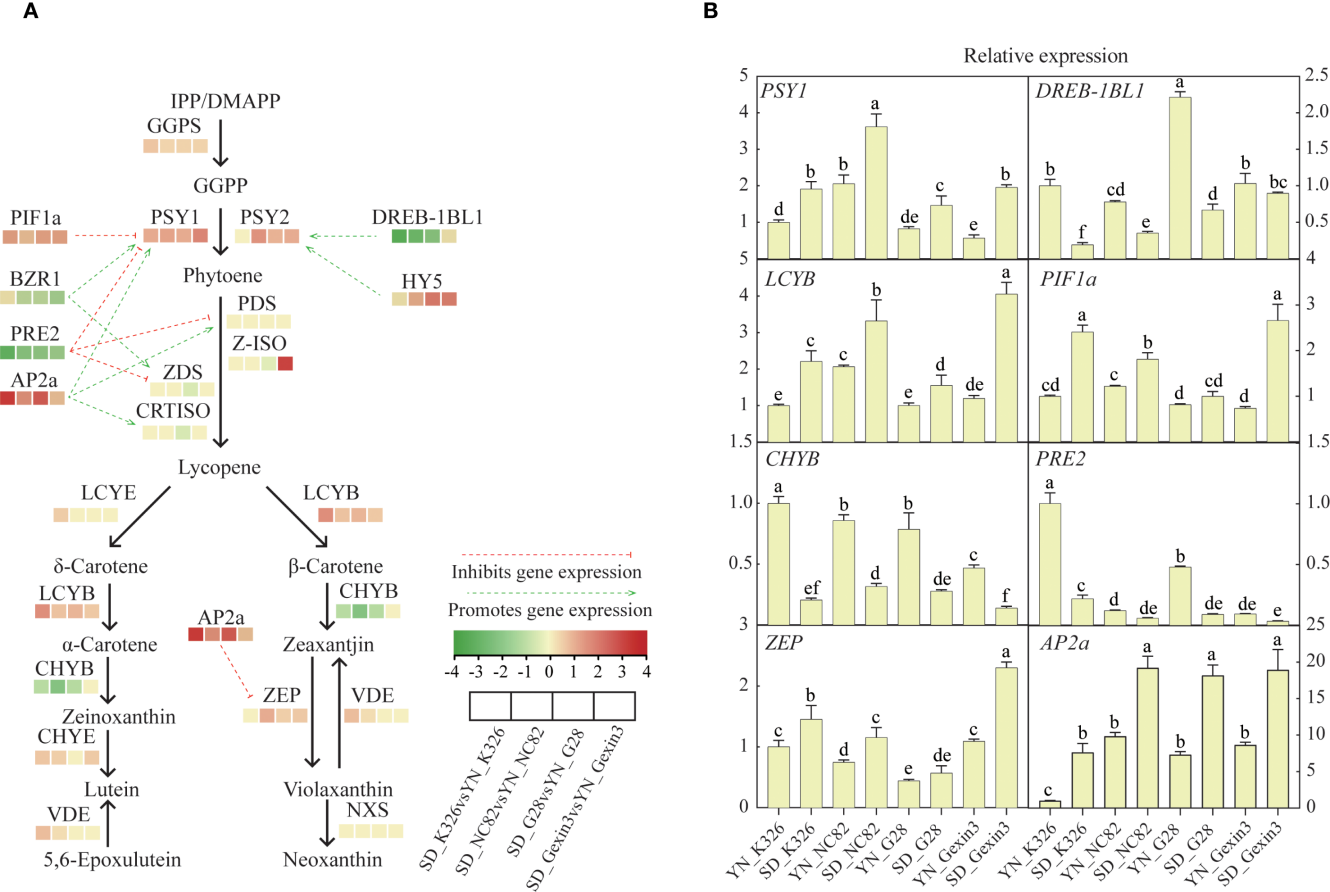
Expression profiling of genes involved in the carotenoid metabolic pathway **(A)** and qRT-PCR analysis of selected genes **(B)**. GGPS, geranyl diphosphate synthase; PSY, phytoene synthase; PDS, phytoene desaturase; Z-ISO, ζ-carotene isomerase; ZDS, ζ-carotene desaturase; CRTISO, carotenoid isomerase; LCYE, lycopene ϵ-cyclase; LCYB, lycopene β-cyclase; CHYB, β-carotene hydroxylase; CHYE, ϵ- cyclase hydroxylase; VDE, violaxanthin de-epoxidase; ZEP, zeaxanthin epoxidase; NXS, neoxanthin synthase. Values labelled with different letters are statistically different from one another using one-way ANOVA (*P* < 0.05).

The structure genes and transcription factors involved in the carotenoid metabolic pathway are crucial for the accumulation of carotenoids (Sun et al., 2018). Interestingly, similar gene expression profiles of the four varieties were observed at the same location (**Figure 3A**). In all of the four varieties, carotenoid biosynthetic genes *GGPS*, *PSY1*, *LCYB*, and transcription factor genes *PIF1a* and *AP2a* showed significantly higher expression in tobacco plants grown in Shandong than those grown in Yunnan. In addition, carotenoid biosynthetic genes *PSY2*, *ZEP* and transcription factor *HY5* were expressed higher in tobacco from Shandong in three of the four varieties. Among these genes, expression of *AP2a* in tobacco grown in Shandong was 9.72-fold, 2.82-fold, 6.11-fold and 1.50-fold higher in K326, NC82, G28 and Gexin3, respectively, than that in tobacco samples from Yunnan. Expression of *PSY1* and *PIF1a* was 2-fold higher in Shandong in all four varieties. On the other hand, other carotenoid-related genes were more expressed in Yunnan respect to Shandong, including biosynthetic gene *CHYB*, transcription factor genes *DREB-1BL1*, *PRE2* and *BZR1*. Among them *PRE2* expression in the Shandong samples was 8.97-fold, 4.46-fold, 3.92-fold and 3.25-fold lower in K326, NC82, G28 and Gexin3 than in the Yunnan samples, respectively. The primary cause of these gene expression changes was the significant difference in ecological environments between the two regions (**Supplementary Table S3**). The elevation of the tobacco-growing area in Yunnan is 1,508 m higher than that in Shandong. The average temperature and average sunlight hours in Shandong were 3.6°C and 0.2h higher, respectively, compared to those in Yunnan. Light triggers changes in genome topology and transcriptional activity. Light-induced genes like *CAB*, *RBCS*, and *GUN5* relocate to the nuclear periphery, correlating with increased expression (Feng et al., 2014). Differential expression of these genes may lead to alterations in the expression levels of a series of downstream genes. Changes in environmental temperature can lead to alterations in plant signaling activities, post-translational modifications, and protein interaction networks (Kaiserli et al., 2018). Furthermore, the soil types also differ between the two regions. The tobacco-growing area in Shandong is characterized by Udic Cambisols, while that in Yunnan features Stagnic Anthrosols (Liu et al., 2022). Differences in soil type affect the diversity and colonization of soil microorganisms, further influencing the development, morphological architecture, and physiological functions of plant roots, thereby collectively impacting plant growth and development (Afzal et al., 2011).

Compared with the influence of environmental factors, the influence of genetic factors over the expression of carotenoid pathway genes was relatively weak. Only a small number of genes showed consistent pattern of changes at different locations but varied among varieties (**Supplementary Figure S4**). Among the four varieties, K326 showed the highest expression of genes *PSY1*, *PSY2*, *PDS*, *Z-ISO*, *ZEP* and *NXS*, and NC82 showed the highest expression genes *LCYE*, *LCYB*, *CHYE*, *VDE* and *VDE*. The results from qRT-PCR analysis confirmed the expression changes of genes involved in carotenoid metabolism (**Figure 3B**).

### 3.4 Carotenoid content analysis of fresh and cured tobacco leaves

**Figure 4:**
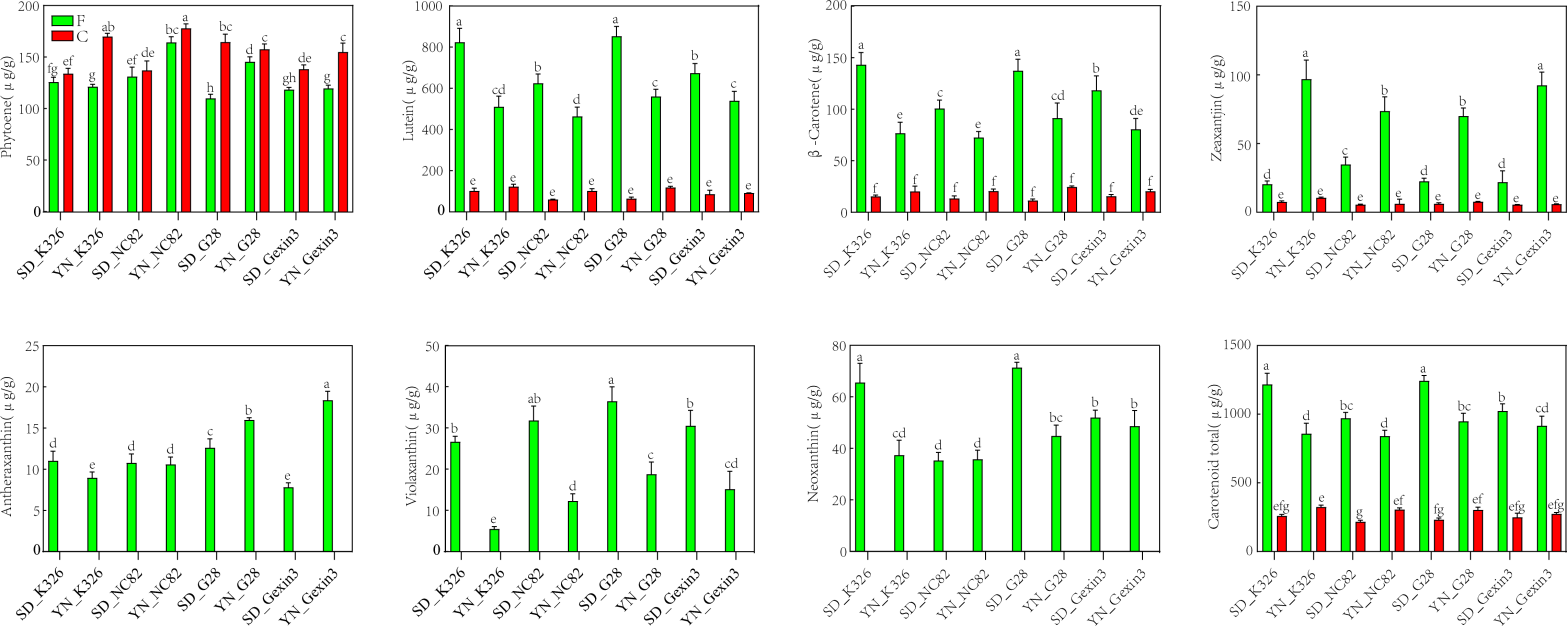
Carotenoid content in fresh (F) and cured (C) tobacco leaves. SD, Shandong; YN, Yunnan. Values labelled with different letters are statistically different from one another using one-way ANOVA (*P* < 0.05).

The content of carotenoids in leaves is one of the major factors affecting tobacco quality. Phytoene, lutein, β-carotene, zeaxanthin, antheraxanthin, violaxanthin, and neoxanthin were detected in fresh tobacco leaves (**Figure 4**). However, only 4 carotenoids were detected in cured tobacco leaves, and these were phytoene, lutein, β-carotene and zeaxanthin (**Figure 4**). This indicates that antheraxanthin, violaxanthin and neoxanthin might be completely degraded to other chemicals by carotenoid-degrading enzymes and high-temperature conditions during the curing process (Fratianni et al., 2013). Interestingly, the content of phytoene did not decrease, but increased after curing, suggesting that there might be also enzymatic reactions during the curing stage (Pola et al., 2020). As the maximum temperature during the yellowing is limited to 37°C, this condition may become suitable for the activation of enzyme-catalysed reactions, including those catalyzed by PSY, favoring the accumulation of phytoene during curing, which needs further experimental verification. In addition, lutein, β-carotene and zeaxanthin decreased significantly after curing. However, no statistically significant difference of these carotenoids was observed between fresh and cured leaves.

It is noteworthy that certain carotenoids in fresh tobacco leaves exhibited discernible consistency among varieties at the same locations. The contents of lutein, β-carotene, violaxanthin, and total carotenoid contents of all four varieties were significantly higher in Shandong than in Yunnan, while zeaxanthin exhibited the opposite trend. Two-way analysis of variance showed that regional variation was the main factor contributing to changes in lutein, β-carotene, violaxanthin, zeaxanthin, and total carotenoid contents in fresh tobacco leaves (**Supplementary Table S4**). Consistently with carotenoid contents, two genes responsible for lutein synthesis, *CHYE* and *VDE*, exhibited significantly higher expression levels in Shandong (**Figure 3**). Furthermore, *LCYB*, which is responsible for β-carotene synthesis, exhibited elevated expression levels in Shandong, whereas the *CHYB*, which catalyzes the conversion of β-carotene to zeaxanthin, demonstrated reduced expression in Shandong compared with Yunnan (**Figure 3**). Moreover, the accumulation of β-carotene in Shandong was greater than in Yunnan, while the accumulation of zeaxanthin in Shandong was lower than that in Yunnan. These results further indicate that environmental factors have a greater influence than genetic factors on the metabolic pathways of tobacco carotenoids.

The degradation of various carotenoids at the curing stage exhibited considerable variability (**Supplementary Table S5**). After curing, the decrease of lutein content ranged from 361.3 to 787.53 μg/g (mean: 537.35 μg/g) respect to fresh leaves, with a content reduction of 75.77% to 92.29% (mean: 84.02%). The decrease of β-carotene content ranged from 56.64 to 127.84 μg/g (mean: 84.94 μg/g), with a content reduction of 71.71 to 91.73% (mean: 80.96%). Zeaxanthin content decreased from 12.9 to 86.55 μg/g (mean: 47.19 μg/g), with a reduction of 62.97 to 93.62% (mean: 82.18%). The content reduction of antheraxanthin, violaxanthin, and neoxanthin was observed to be 7.83 to 18.4 μg/g (mean: 12.03 μg/g), 5.55 to 36.56 μg/g (mean: 22.22 μg/g), and 35.33 to 71.37 μg/g (mean: 48.84 μg/g), respectively, with 100% reduction. Lutein exhibited the greatest degree of degradation, particularly in SD_G28, with a reduction of up to 787.53 μg/g. The greatest decline in total carotenoid content (995.79 μg/g, 80.07%) was observed in SD_G28. The degradation of carotenoids could result in production of aroma substances, which are conducive in promoting the quality of tobacco (Enzell, 1985). To gain insight into the changes in the degradation products of carotenoids, we proceeded to measure the aroma compounds present in tobacco leaves before and after curing.

### 3.5 Aroma substance content analysis in fresh and cured tobacco leaves

The composition and level of aroma substances is one of the most direct and reliable indicators of tobacco leaf quality (Nedeltcheva-Antonova et al., 2016; Wu et al., 2013). In this study, we found that the aroma substances in tobacco changed significantly before and after curing (**Supplementary Table S6**). A total of 50 aroma substances were identified in fresh tobacco leaves, comprising 14 CDPs, 1 chlorophyll degradation product, 17 browning reaction products, 6 phenylalanine degradation products, 2 cembrane degradation products, and 10 other aroma substances. A total of 72 aroma substances were identified in cured tobacco leaves, comprising 23 carotenoid degradation products, 1 chlorophyll degradation product, 24 browning reaction products, 8 phenylalanine degradation products, 3 cembrane degradation products, and 13 other aroma substances. One single aroma substance was identified exclusively in fresh tobacco leaves, while 23 aroma substances were exclusively detected in cured tobacco leaves. 49 aroma substances were identified in both fresh and cured tobacco leaf samples. The majority of these substances exhibited a similar pattern of changes during curing, with 26 aroma substances demonstrating a significant increase after curing and 6 aroma substances exhibiting a significant decrease. The highest content of these aroma substances is neophytadiene, with concentrations in fresh tobacco leaves ranging from 389.34 to 1292.29 ng/g and in cured tobacco leaves ranging from 1,237.62 to 2,042.75 ng/g. This substance is a degradation product of chlorophyll, which directly affects the flavor and aroma of tobacco leaves (Chao et al., 2022).

**Figure 5:**
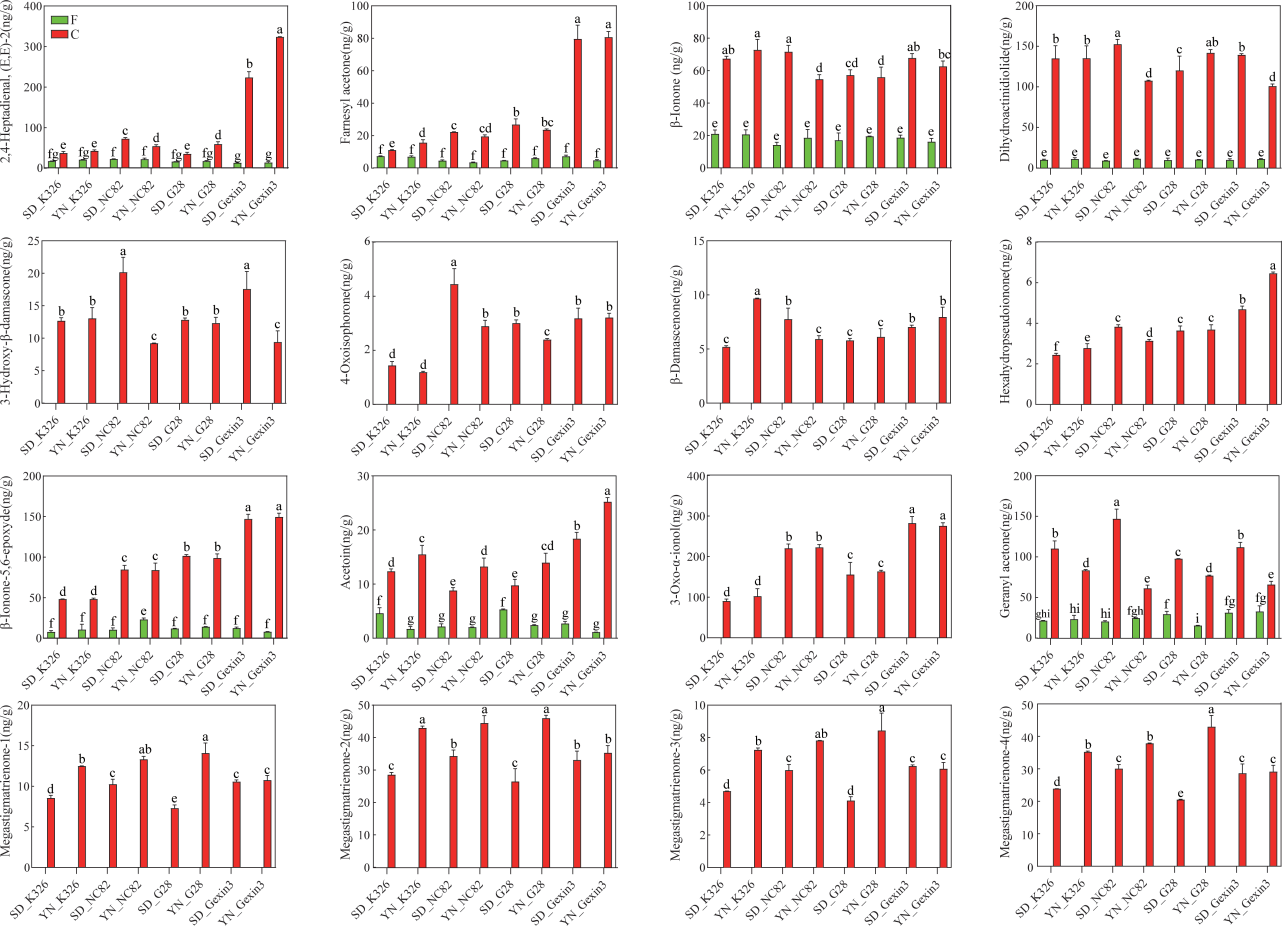
Carotenoid degradation products in fresh (F) and cured (C) tobacco leaves. SD, Shandong; YN, Yunnan. Values labelled with different letters are statistically different from one another using one-way ANOVA (*P* < 0.05).

During the process of curing, carotenoids can be degraded by enzymatic and non-enzymatic reactions to generate CDPs. The CCD enzymes play a crucial role in the carotenoid degradation pathway. CCDs facilitate the degradation of carotenoids, resulting in the formation of β-ionone, cyclocitral, dihydroactinidiolide, and other compounds (Bouvier et al., 2003; Schwartz et al., 2004; Sun et al., 2008). In plants, lipoxygenases (LOXs) catalyze the oxidation of polyunsaturated fatty acids, such as linoleic and linolenic acid. Free radicals generated during the intermediate steps of this process mediate the nonspecific oxidative degradation of carotenoid pigments. This LOX-induced co-oxidation reaction cleaves carotenoids and yields a variety of apocarotenoid volatiles, including β-ionone, β-cyclocitral, β-ionone-5,6-epoxide, and dihydroactinidiolide (Enzell, 1985; Liang et al., 2021; Wache et al., 2006). During the curing stage, LOXs may first act on polyunsaturated fatty acids in tobacco leaves, and the resulting free radicals then promote the degradation of carotenoids into various aroma compounds. The non-enzymatic degradation of carotenoids is primarily observed in the final stages of curing, with elevated temperatures facilitating the cleavage of carbon chains, leading to the formation of β-damascone, isophorone, and other compounds (Bezman et al., 2005; Rios et al., 2008). In this study, we found that 14 CDPs were detected in both fresh and cured tobacco leaves. Among these, 2,4-heptadienal, (E,E)-2, farnesyl acetone, β-ionone, dihydroactinidiolide, β-ionone-5,6-epoxyde, acetoin, and geranyl acetone exhibited a notable increase following the curing process. Conversely, 6-methyl-2-heptanone demonstrated a decline in concentration (**Figure 5**, **Supplementary Table S6**). It is noteworthy that 2,4-heptadienal, (E,E)-2 and farnesyl acetone exhibited particularly elevated levels in cured leaves of Gexin3, exceeding three folds the concentrations observed in the other three varieties. This observation suggests that these compounds can highlight the cultivars specificity of Gexin3. 3-oxo-α-ionol, 3-hydroxy-β-damascone, 4-oxoisophorone, β-damascenone, hexahydropseudoionone, megastigmatrienone-1, megastigmatrienone-2, megastigmatrienone-3, and megastigmatrienone-4 were identified exclusively in cured tobacco leaves (**Figure 5**), suggesting that these compounds are potentially produced exclusively by degradation of carotenoids from fresh tobacco leaves. Two-way analysis of variance revealed that location variation was the primary factor influencing changes in the content of geranyl-acetone, acetoin, megastigmatrienone-1, megastigmatrienone-2, megastigmatrienone-3, and megastigmatrienone-4 in cured tobacco leaves; variety variation was the primary factor influencing changes in the content of 2,4-heptadienal, (E,E)-2, farnesyl acetone, 4-Oxoisophorone, Hexahydropseudoionone, β-ionone-5,6-epoxide, and 3-oxo-α-ionol in cured tobacco leaves; the combined effects of location and variety are the primary factors influencing the changes in the contents of β-ionone, 3-hydroxy-β-damascone, β-damascenone, and dihydroactinidiolide in cured tobacco leaves (**Supplementary Table S7**). It is noteworthy that geranyl aceton and acetoin in cured tobacco leaves exhibited clear consistency in the four varieties from the same location. The geranyl aceton content of all four varieties was significantly higher in Shandong than in Yunnan, whereas the acetoin content exhibited the opposite trend. Furthermore, megastigmatrienone-1, megastigmatrienone-2, megastigmatrienone-3, and megastigmatrienone-4 in K326, NC82, and G28 also demonstrated consistency in the same location. In contrast, β-ionone-5,6-epoxyde and 3-oxo-α-ionol exhibited varietal specificity, and changes in the cultivation environment did not result in variations in the levels of aroma substances. While the carotenoids of fresh tobacco leaves did not demonstrate any particular specificity with regard to the variety, some CDPs of cured tobacco leaves exhibited such specificity. This indicates that the formation of aroma substances in cured tobacco leaves is closely associated with curing characteristics. Thus, it can be inferred that both environment and genotype have an effect on the formation of CDPs in cured tobacco leaves.

## 4 Conclusions

In this study we investigated the effects of environmental and genetic factors on the content of CDPs in cured tobacco leaves. Transcriptome analysis revealed that differentially expressed genes (DEGs) between Shandong and Yunnan were enriched in the carotenoid biosynthetic pathway, with key genes (e.g., *CHYE*, *VDE*, *LCYB*, *CHYB*) and transcription factors (e.g., *PIF1a*, *AP2a*) showing location-specific expression patterns. These expression differences directly affected carotenoid profiles: Shandong-grown tobacco accumulated more lutein and β-carotene, whereas Yunnan tobacco contained higher zeaxanthin. During the curing stage, carotenoids were degraded into aroma compounds. Although the gene expression profiles and multiple carotenoids in fresh leaves showed similarity in the same location, the CDPs of cured tobacco leaves showed more complex patterns of changes, with some aroma substances showing varietal specificity. Among the detected CDPs, acetoin, geranyl acetone, and megastigmatrienone were primarily affected by environmental factors, whereas β-ionone-5,6-epoxyde and 3-oxo-α-ionol were predominantly influenced by genetic factors. Therefore, the formation of CDPs in cured tobacco leaves is influenced by both environmental and genetic factors. In summary, this study systematically reveals the cascade regulatory relationship among “gene expression – carotenoid accumulation – aroma formation”. This finding provides a theoretical basis for improving the aroma quality of tobacco leaves through strategies that combine environmental regulation and variety selection.

## Data Availability

The data presented in the study are deposited in the NCBI repository, accession number PRJNA1294826. This data can be found here: https://www.ncbi.nlm.nih.gov/sra/PRJNA1294826.
