# Supplementary material for: Transcriptomic and metabolomic analysis of carotenoid metabolism and accumulation of carotenoid-derived products in tobacco leaves before and after curing
Source: Front Plant Sci. 2025 Oct 10;16:1671379. doi: 10.3389/fpls.2025.1671379 (PMC12549664; doi:10.3389/fpls.2025.1671379)
Supplement: Supplementary file 1 [file Table1.docx]

Supplementary Material


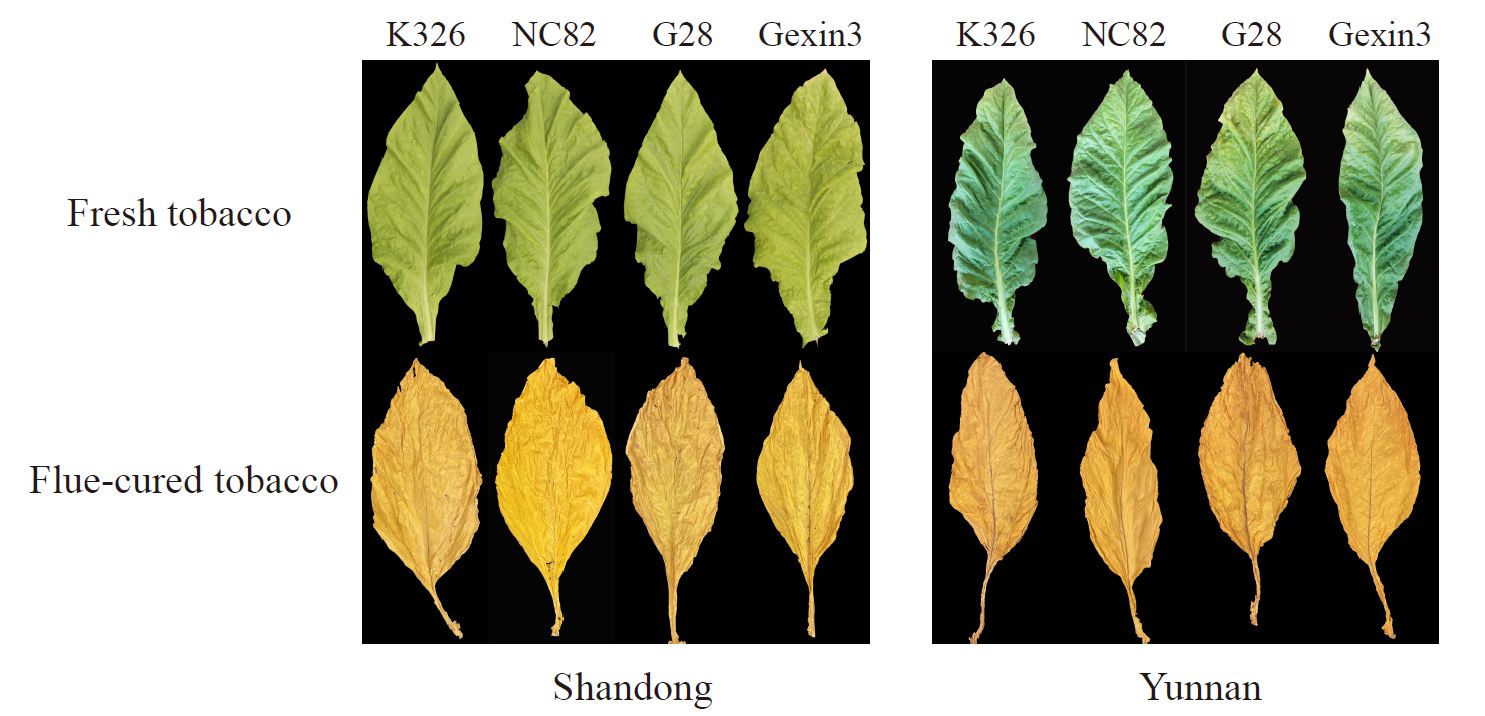


Fig S1. Samples of fresh and flue-cured tobacco leaves collected from four varieties (K326, NC82, G28 and Gexin3) and two locations (Shandong and Yunnan).


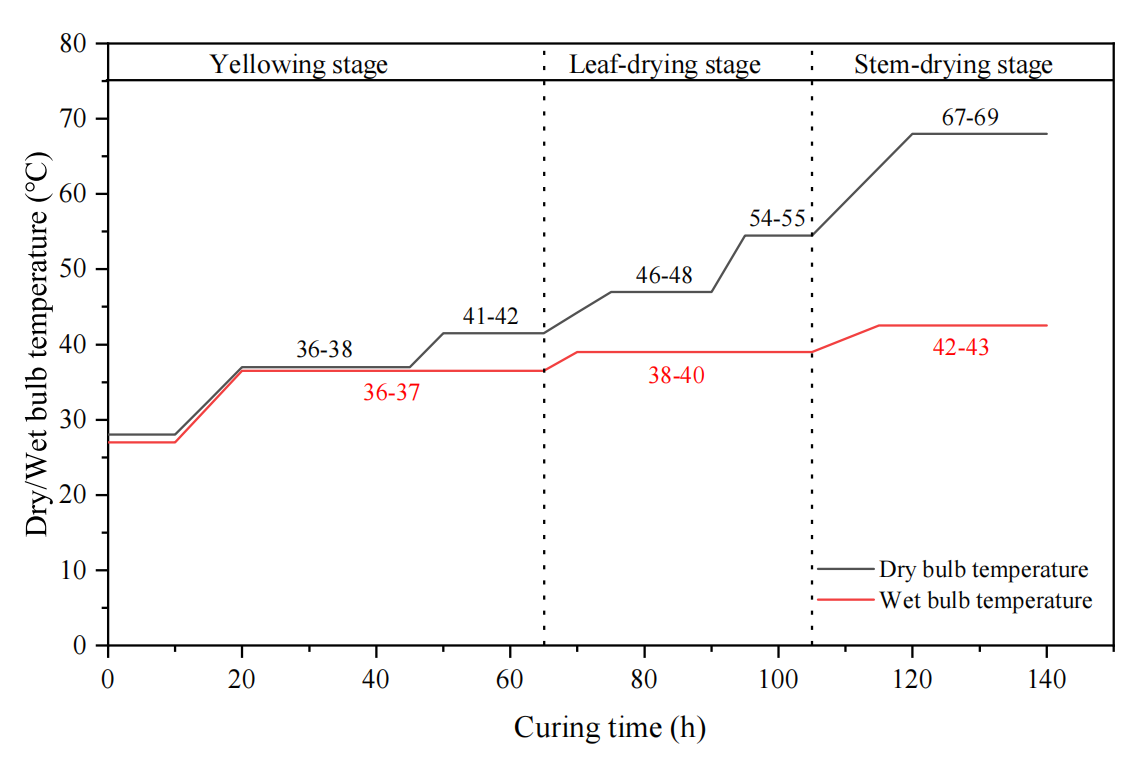


Fig S2. Three-stage flue-curing technology.


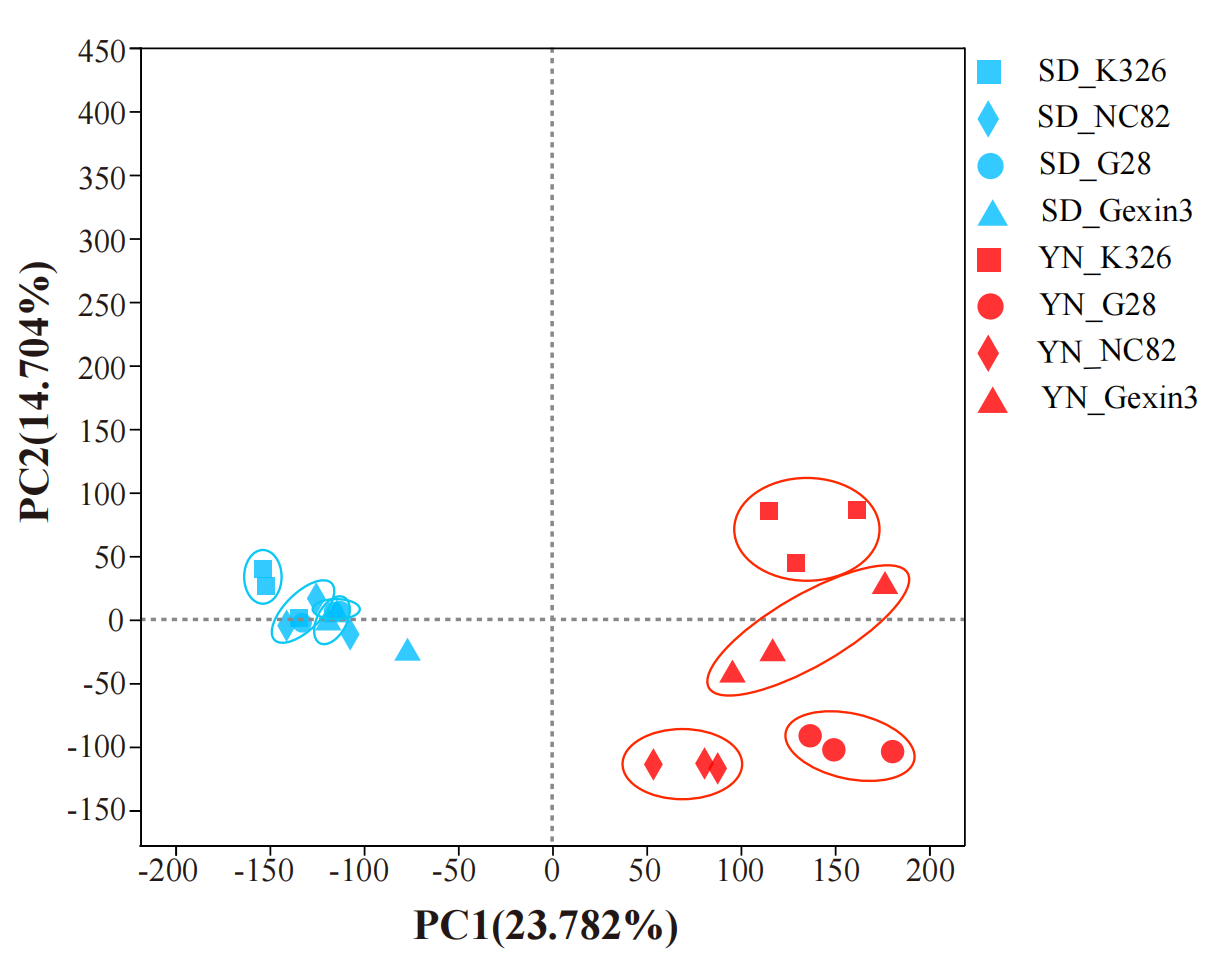


Fig S3. Principal component analysis (PCA) of transcriptomic data. The circles indicate the 95% confident regions.


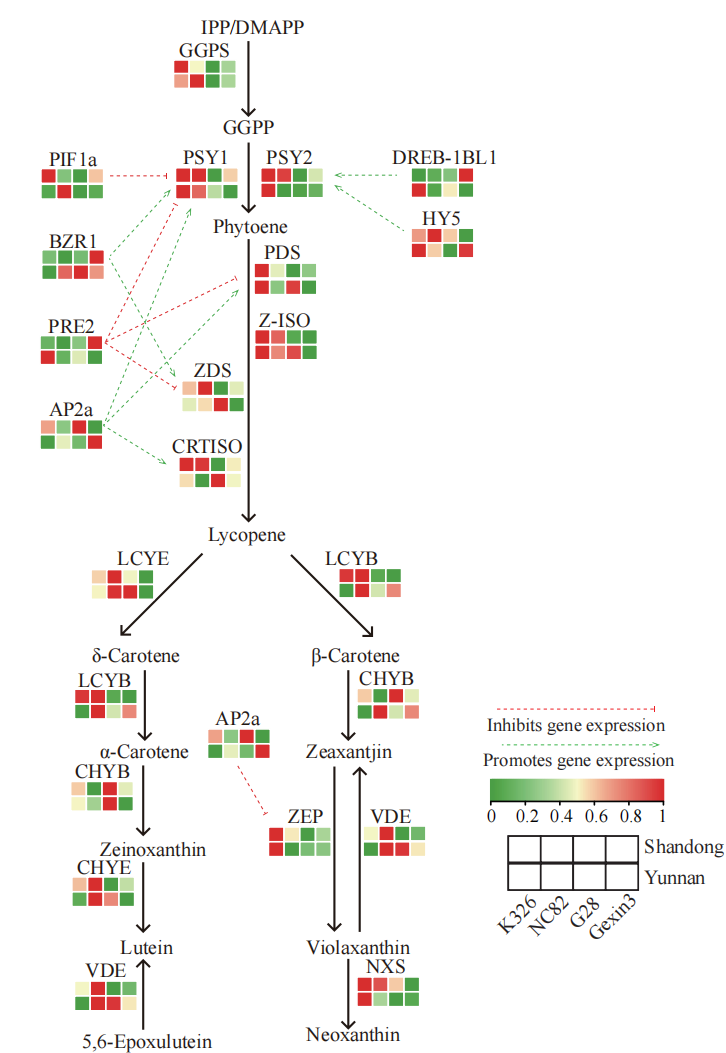


Fig S4. Expression profiling of genes involved in the carotenoid metabolic pathway. Expression levels were scaled into a relative expression scale using z-score transformation. Red represents up-regulation and green indicates down-regulation.
